# Superstrong, superstiff, and conductive alginate hydrogels

**DOI:** 10.1038/s41467-022-30691-z

**Published:** 2022-05-31

**Authors:** Donghwan Ji, Jae Min Park, Myeong Seon Oh, Thanh Loc Nguyen, Hyunsu Shin, Jae Seong Kim, Dukjoon Kim, Ho Seok Park, Jaeyun Kim

**Affiliations:** 1 School of Chemical Engineering, Sungkyunkwan University (SKKU), Suwon, 16419 Republic of Korea; 2 Department of Health Sciences and Technology, Samsung Advanced Institute for Health Sciences & Technology (SAIHST), Sungkyunkwan University (SKKU), Suwon, 16419 Republic of Korea; 3 Biomedical Institute for Convergence at SKKU (BICS), Sungkyunkwan University (SKKU), Suwon, 16419 Republic of Korea; 4 Institute of Quantum Biophysics (IQB), Sungkyunkwan University (SKKU), Suwon, 16419 Republic of Korea

**Keywords:** Gels and hydrogels, Mechanical properties, Polymers

## Abstract

For the practical use of synthetic hydrogels as artificial biological tissues, flexible electronics, and conductive membranes, achieving requirements for specific mechanical properties is one of the most prominent issues. Here, we demonstrate superstrong, superstiff, and conductive alginate hydrogels with densely interconnecting networks implemented via simple reconstructing processes, consisting of anisotropic densification of pre-gel and a subsequent ionic crosslinking with rehydration. The reconstructed hydrogel exhibits broad ranges of exceptional tensile strengths (8–57 MPa) and elastic moduli (94–1,290 MPa) depending on crosslinking ions. This hydrogel can hold sufficient cations (e.g., Li^+^) within its gel matrix without compromising the mechanical performance and exhibits high ionic conductivity enough to be utilized as a gel electrolyte membrane. Further, this strategy can be applied to prepare mechanically outstanding, ionic-/electrical-conductive hydrogels by incorporating conducting polymer within the hydrogel matrix. Such hydrogels are easily laminated with strong interfacial adhesion by superficial de- and re-crosslinking processes, and the resulting layered hydrogel can act as a stable gel electrolyte membrane for an aqueous supercapacitor.

## Abstract (web summary)

Specific mechanical properties are one of the most important issues for application of synthetic hydrogels as biological tissue, flexible electronics or in conductive membranes. Here, the authors demonstrate that a reconstruction process consisting of anisotropic densification of pre-gel and subsequent ionic crosslinking and rehydration leads to strong, stiff, and conductive alginate hydrogels with densely interconnecting networks.

## Introduction

Unlike metals, ceramics, or typical plastics, hydrogels are semi-solid with high water content. Because hydrogels are generally viscoelastic, shapable, and ionically conductive, they can be utilized in various areas, such as artificial biological tissues, soft robots, flexible backbones of bioelectronics, conductive membranes, and solid gel electrolytes of energy storage devices (e.g., batteries)^1–8^. For bio-applications, a mechanical match between the hydrogel and surrounding tissues should be considered to minimize deformation, irritation, immune response, and electrical decoupling^8–10^. However, the strength and stiffness of existing synthetic hydrogels remain significantly lower than those of several biological tissues, in particular, load-bearing connective tissues such as ligaments, tendons, cartilages, and blood vessels with tensile strengths of 0.1–200 MPa and elastic moduli of 0.1–3 GPa^11–15^. For use as a solid-like gel electrolyte membrane for energy storage devices, the hydrogel should possess superior mechanical performance, particularly tensile strength and elastic modulus. Because this electrolyte membrane acts as a separator between electrodes, strong and stiff hydrogels can maintain its mechanical integrity, when subjected to external impacts and electrode surface reactions that can cause internal short circuits. In addition to the mechanical performance, sufficient ionic conductivity of the electrolyte is also essential for decreasing both the electrolyte resistance and the voltage losses between the electrodes that can cause a low charge-discharge rate^16^. To date, practical use of the existing hydrogel for such applications has been limited owing to its weak mechanical properties (a few kilopascals’ strengths and moduli)^17–19^. Therefore, developing hydrogels possessing superior strength, stiffness, and conductivity is a prominent challenge.

Alginate (Alg), a natural polysaccharide polymer, is one of the most widely used polymers for hydrogel fabrication because of good processability with ionic crosslinking, environmental sustainability, biocompatibility (low toxicity), and controllable biodegradability^20,21^. Despite these advantages, the inferior mechanical properties limit the utilization of Alg hydrogels. Several methods have been proposed for addressing this problem. The mechanical properties of Alg hydrogels are typically improved by increasing the Alg chain length or Alg concentration of a precursor solution^22–24^. These approaches generally need a highly viscous precursor Alg solution, which causes difficulties in handling and obtaining uniform hydrogels. An additional introduction of strong covalent crosslinking can substantially enhance the stiffness of the Alg hydrogel; however, the hydrogel generally becomes brittle and the strength declines^25^. Strong and stiff reinforcements such as particles and fibers are also often mixed with the Alg solution to obtain mechanically reinforced Alg hydrogels. Despite that, the mechanical performances of the existing composite hydrogels are not sufficient to afford strengths and elastic moduli of MPa-to-GPa magnitude^26–29^. Another method, a drawing method, which is a post-process to increase the Alg concentration and crosslinking density of preformed hydrogels, produces a significant mechanical performance enhancement with a form of fiber/thread-like hydrogels^30–32^. However, they are apparently not suitable for manufacturing large-sized hydrogels with a uniform shape and homogeneous mechanical properties because an additional step, such as weaving, should be included for fabricating large-sized hydrogels.

**Fig. 1:**
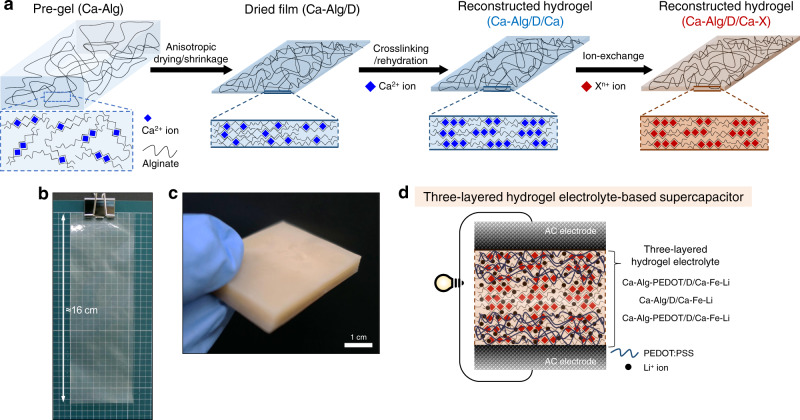
Schematics of fabrication for reconstructed Alg hydrogel and mechanically robust aqueous supercapacitor. **a** Fabrication steps of the reconstruction method: anisotropic drying/shrinkage of Ca–Alg pre-gel and crosslinking/rehydration in Ca^2+^ solution. To futher enhance the hydrogel mechanical performance, an ion-exchange step from Ca^2+^ to X^n+^ (Ba^2+^, Al^3+^, or Fe^3+^) is additionally introduced. **b**, **c** Photographs showing a reconstructed hydrogel with a large area and a thick bulky hydrogel by lamination, respectively. **d** Schematic illustration of a mechanically robust aqueous supercapacitor composed of activated carbon (AC) electrodes and three-layered hydrogel electrolyte.

Herein, we report superstrong, superstiff, and conductive Alg hydrogels implemented via a simple fabrication method (Fig. 1a). The proposed method consists of two steps, anisotropic drying/shrinkage of a pre-gel attached on the flat substrate and additional crosslinking with rehydration in an ionic solution, which is designated as a reconstruction method. The anisotropic drying/shrinkage and subsequent crosslinking/rehydration processes produce densely interconnected Alg networks, significantly enhancing hydrogel strength and stiffness. The simplicity of the process facilitates the production of hydrogels with a large area and thick bulky hydrogels (Fig. 1b, c). The resultant hydrogel possesses variable tensile strengths and elastic moduli in the MPa-to-GPa range, depending on the crosslinking ions (Ca^2+^, Ba^2+^, Al^3+^, or Fe^3+^ in this study); these features have not been achieved previously in hydrogel systems. Moreover, the sufficient water content of the hydrogel allows high cation dissolution (e.g., Li^+^) within the gel without compromising the hydrogel mechanical performance. This hydrogel exhibits a high ionic conductivity (2.2 mS/cm) sufficient for its potential use as a solid gel electrolyte membrane for energy storage devices. Furthermore, we can fabricate an ionic-/electrical-conductive hydrogel by mixing an electrically conducting poly(3,4-ethylenedioxythio phene):poly(styrene sulfonate) (PEDOT:PSS) in the Alg matrix. Such hydrogels are easily laminated with strong interfacial adhesion through superficial de-crosslinking and re-crosslinking processes. A three-layered hydrogel composed of PEDOT-contained top and bottom layers demonstrates potential usability as a stable gel electrolyte membrane for an aqueous supercapacitor (Fig. 1d).

## Results and discussion

### Design of hydrogels with densely interconnected Alg networks

To date, two methods have been conventionally used for fabricating Alg hydrogels. The first method involves soaking an Alg solution in a Ca^2+^ solution, resulting in an externally gelated hydrogel denoted as Alg/Ca (Supplementary Fig. 1a). Due to fast ionic crosslinking in the Ca^2+^ solution, the Alg solution is immediately agglomerated, forming an uneven Alg/Ca hydrogel; thus, it is difficult to evaluate its mechanical performance. The second method involves mixing an Alg solution with CaSO_4_ slurry that slowly dissociates Ca^2+^ ions in the Alg solution, resulting in an internally gelated hydrogel denoted as Ca–Alg (Supplementary Fig. 1b). Although Ca–Alg has more even physical and mechanical properties throughout the gel, its mechanical properties are weak due to a limited amount of CaSO_4_ used to allow slow and uniform gelation. Because these Alg hydrogels contain much water in the sparse Alg networks with large pores (Supplementary Fig. 1c, d), the strength and stiffness could not reach the MPa-to-GPa range.

**Fig. 2:**
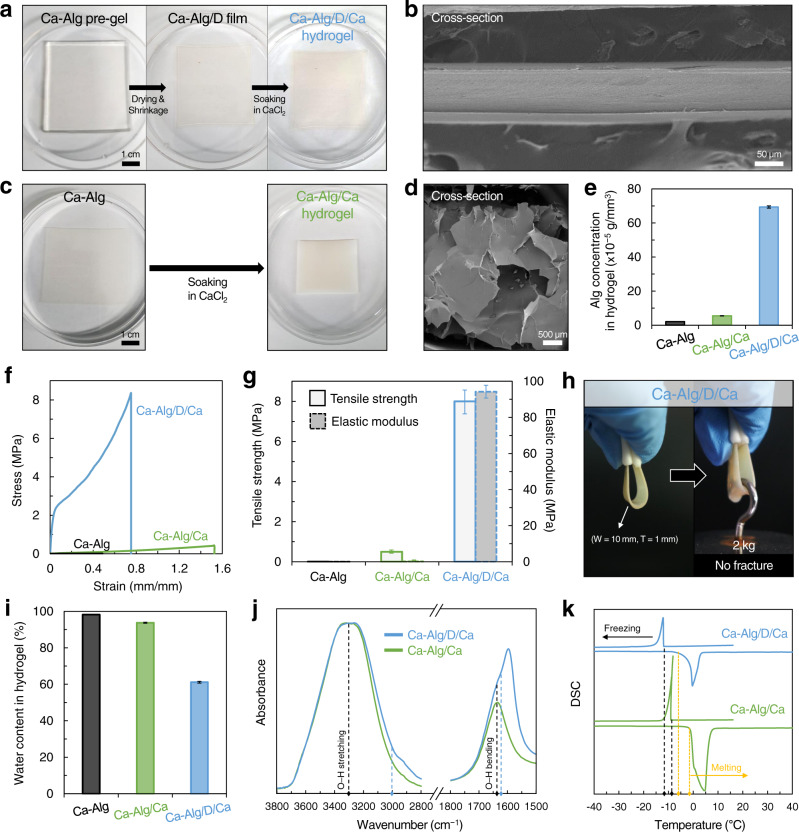
Fabrication and mechanical properties of reconstructed hydrogel. **a** Photographs showing the hydrogel sample at each step, anisotropic drying/shrinkage and crosslinking/rehydration. **b** Cross-sectional scanning electron microscope (SEM) image of the resulting reconstructed Ca–Alg/D/Ca hydrogel. **c** Photographs showing Ca–Alg and Ca–Alg/Ca hydrogels, respectively, before and after soaking in CaCl_2_ solution. **d** Cross-sectional SEM image of Ca–Alg/Ca hydrogel. **e** Alginate concentration in each hydrogel. **f** Stress–strain curve and **g** tensile strength and elastic modulus of Ca–Alg, Ca–Alg/Ca, and Ca–Alg/D/Ca hydrogels. **h** Photographs representing the mechanical robustness of Ca–Alg/D/Ca hydrogel. **i** Water content in each hydrogel. **j** Fourier-transform infrared (FTIR) spectra and **k** Differential scanning calorimetry (DSC) curves of Ca–Alg/Ca and Ca–Alg/D/Ca hydrogels, exhibiting differences of interaction degrees between the sparse and densified polymer networks. SEM images were obtained after lyophilization. Error bars correspond to standard deviations.

To address such problems and fabricate strong, stiff Alg hydrogels, we devised a reconstruction method that densifies the polymer networks and fastens the closed polymer chains. This method is composed of two processes: “anisotropic drying and shrinkage of a pre-gel (anisotropic drying/shrinkage)” and “additional crosslinking with rehydration in an ionic solution (crosslinking/rehydration)” (Fig. 1a). As a concrete example, a Ca–Alg pre-gel with 3 mm thickness, which was first weakly crosslinked by CaSO_4_, was anisotropically dried and shrunk on a flat substrate almost without a decrease in the width and area (Fig. 2a). The resulting dried film (Ca–Alg/D) of 45 μm thickness was soaked in 100 mM CaCl_2_ solution to obtain a rehydrated, densely Ca-crosslinked hydrogel (Ca–Alg/D/Ca) with 90 μm thickness. The thicker pre-gel (e.g., 10 mm thickness) could be also used to produce a thicker reconstructed hydrogel (e.g., 300 μm thickness). When the pre-gel is thicker than 10 mm, the *x*–*y* plane shrinkage is unavoidable, resulting in an inhomogeneous sample. The thickness of the final Ca–Alg/D/Ca hydrogel was considerably reduced (3% of that of Ca–Alg) because of the highly interconnected network, where the hydrogel exhibited a uniform and compact structure even after lyophilization (Fig. 2b). The dense Ca^2+^ crosslinking in Alg networks enables the Ca–Alg/D/Ca hydrogel to maintain its shape in pure water, which contrasts with a complete dissociation of limitedly crosslinked Ca–Alg/D (Supplementary Fig. 2). When the Ca–Alg was isotropically dried in the air, rather than anisotropically dried/shrunk on the substrate, a uniform hydrogel could not be obtained (Supplementary Fig. 3).

The subsequent soaking of Ca–Alg/D dried film in CaCl_2_ solution resulted in homogeneous Ca^2+^ crosslinking within the densified network (Supplementary Fig. 4). In a typical preparation, a fully crosslinked Ca–Alg/D/Ca hydrogel with 90 μm thickness was obtained in 5 min (Supplementary Fig. 5). The Alg chains and networks were in an amorphous phase regardless of the Ca^2+^ crosslinking (Supplementary Fig. 6). A simple lamination method is utilized to fabricate a thick bulky Ca–Alg/D/Ca hydrogel using the 90 μm-thick reconstructed hydrogels (Supplementary Fig. 7). For the lamination, the surface of thin Ca–Alg/D/Ca hydrogel (90 μm) was shortly treated with ethylenediaminetetraacetic acid (EDTA) solution, and the EDTA-treated hydrogels were stacked under gentle pressure for a few minutes. Because EDTA sequestered Ca^2+^ from the superficial Alg networks and softened the hydrogel surface, the resulting free Alg chains on the softened surface were gradually re-crosslinked by Ca^2+^ ions diffused out from the hydrogel insides; the Ca–Alg/D/Ca hydrogels contained surplus Ca^2+^ ions because they were obtained after soaked in the CaCl_2_ solution. As a result, the interface of the adjacent hydrogels bonded quickly by Alg chains re-crosslinking without any glues. The lap-shear test confirmed the strong interfacial adhesion between the Ca–Alg/D/Ca hydrogels (re-crosslinked interface after the EDTA treatment) (Supplementary Fig. 8a). Despite the mechanical robustness of the reconstructed hydrogel, strong interfacial adhesion caused a rupture of the hydrogel itself first. In addition, the adhesive shear strength was larger than at least 420 kPa, which is superior to that of the exiting hydrogel adhesives and polymeric tissue adhesives^33,34^, indicating that this lamination allows stable adhesion between the reconstructed hydrogels. Therefore, the resulting bulky hydrogel was mechanically robust and bendable without interfacial detachment and separation (Supplementary Fig. 8b).

The reconstruction method contrasts with direct soaking of the Ca–Alg pre-gel in CaCl_2_ solution without the anisotropic drying/shrinkage process (Ca–Alg/Ca hydrogel, Fig. 2c). Ca–Alg/Ca possesses a porous structure (Fig. 2d) due to the sparse Alg network with high water content even after soaking in CaCl_2_. Such structural differences between the Ca–Alg/D/Ca and Ca–Alg/Ca hydrogels are attributed to the difference in the Alg concentration in hydrogel (the weight of Alg per unit volume of hydrogel) (Fig. 2e). Ca–Alg/D/Ca exhibited an incomparably high polymer concentration with highly interconnected compact networks (Fig. 2b).

The importance of pre-gel formation for obtaining a uniform hydrogel was also confirmed. When the Alg solution, rather than the Ca–Alg pre-gel, was anisotropically dried/shrunk and soaked in CaCl_2_ solution, a wrinkled and rolled-up hydrogel was obtained (Supplementary Fig. 9). The difference between the wrinkled hydrogel obtained from the Alg/D film and the flat hydrogel obtained from the Ca–Alg/D film is attributed to the difference in the drying process. While the interconnected Alg chains (Ca–Alg pre-gel) can hardly be accumulated at the gel/air interface during the water evaporation, the relatively free-movable Alg chains in Alg solution can be easily accumulated at the solution/air interface^35,36^. The difference in surface hardness between the top (toward the air) and the bottom (toward the substrate) of the dried Alg/D/Ca sample confirmed such a phenomenon (Supplementary Fig. 9c). In contrast to no difference in the dried Ca–Alg/D/Ca surface hardness, there is an approximately 10 % difference in the dried Alg/D/Ca surface hardness.

### Enhanced mechanical properties of reconstructed hydrogel

The reconstruction of the polymer network significantly enhanced the hydrogel mechanical performance (Fig. 2f–h). Although the additional Ca-crosslinking (Ca–Alg/Ca) improved the mechanical properties compared to the initial hydrogel (Ca–Alg), both the tensile strength and elastic modulus were still low at approximately a few hundred kilopascals. On the other hand, the tensile strength and elastic modulus of the reconstructed hydrogel (Ca–Alg/D/Ca) were significantly high as the MPa level (tensile strength of 8 MPa and elastic modulus of 94 MPa) (Fig. 2g), indicating the densely crosslinked compact Alg networks contributed to the dramatical enhancement. This strong, stiff, yet flexible Ca–Alg/D/Ca hydrogel withstood two kg-weight (Fig. 2h). Despite its high water content (61 wt%) (Fig. 2i), the tensile strength and elastic modulus of Ca–Alg/D/Ca were 1640- and 7220-fold higher, respectively, than those of the Ca–Alg. The reconstructed hydrogels obtained after the crosslinking/rehydration longer than 30 min in Ca^2+^ solution possessed similar mechanical properties (Supplementary Fig. 10), which is in line with the result of the homogenous crosslinking completed in 5 min in Ca^2+^ solution (Supplementary Fig 5).

These mechanically substantial enhancements are attributed to the increased interactions in the densified polymer networks, which were identified by Fourier-transform infrared (FTIR) and differential scanning calorimetry (DSC) analyses. Compared to the peaks observed in Ca–Alg/Ca, the peak broadening of the O–H stretching at 3000–3700 cm^−1^ and the O–H bending at 1600–1700 cm^−1^ in Ca–Alg/D/Ca were observed (Fig. 2j and Supplementary Fig. 11); the COO^−^ stretching peaks at 1595 cm^−1^ was amplified due to the densified polymer networks^37–41^. Because the number of hydrogen bonding between polymer chains and between polymer chains/water molecules was increased in the densified polymer networks of Ca–Alg/D/Ca hydrogel, the O–H bond was easier to be stretched and bent; therefore, these O–H bond was represented as the increase in the peak intensity of O–H stretching and O–H bending at low wavenumber ranges, 3000–3300 cm^−1^ and 1625–1635 cm^−1^, respectively. A clear decrease in freezing and melting points of water in the Ca–Alg/D/Ca hydrogel compared to that of the Ca–Alg/Ca hydrogel supports the above results (Fig. 2k). The water confined in the densified polymer networks of Ca–Alg/D/Ca hydrogel froze at a temperature near −13 °C (left black-line) and the frozen water thawed at a temperature near −6 °C (left yellow-line); these temperatures were lower than those of water in the sparse polymer networks of Ca–Alg/Ca (freezing near −9 °C of right black-line and thawing near −1 °C of right yellow-line).

### Additional mechanical enhancement of reconstructed hydrogel via ion exchange

**Fig. 3:**
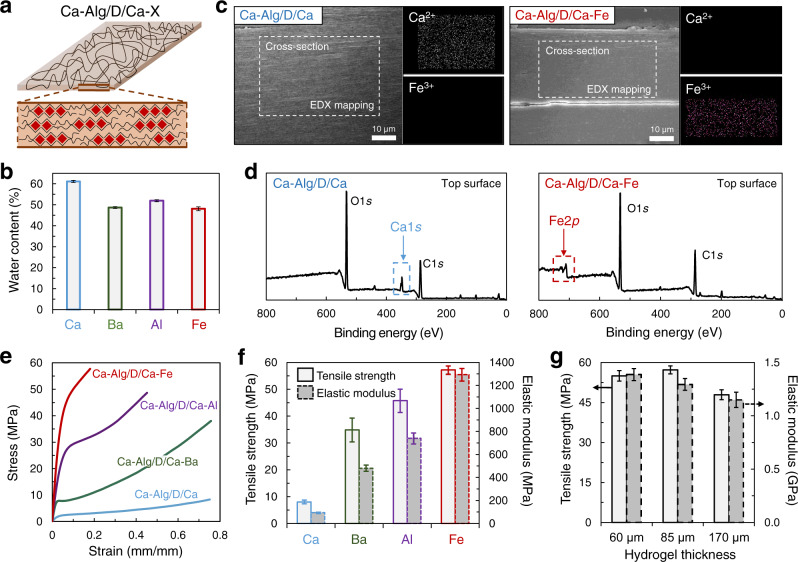
Enhanced mechanical properties of the reconstructed hydrogels crosslinked by various cations. **a** Illustration of secondary-ion-crosslinked Ca–Alg/D/Ca–X hydrogels through the exchange from Ca^2+^ ions with other cations by soaking in BaCl_2_, AlCl_3_, or FeCl_3_ solution. **b** Water content in the resulting hydrogels. **c** Energy-dispersive X-ray spectroscopy (EDX) mapping of the cross-section and **d** X-ray photoelectron spectroscopy (XPS) profiles of the upper surface of Ca–Alg/D/Ca and Ca–Alg/D/Ca–Fe. **e** Stress-strain curves and **f** tensile strengths and elastic moduli of the reconstructed hydrogels. **g** Tensile strengths and elastic moduli of Ca–Alg/D/Ca–Fe with different thicknesses. Error bars correspond to standard deviations.

The mechanical properties of the reconstructed hydrogels were further enhanced by using different secondary crosslinking ions, Ba^2+^, Al^3+^, or Fe^3+^ (Fig. 3a). Ba, Al, or Fe-crosslinked reconstructed hydrogels (Ca–Alg/D/Ca–X, X = Ba, Al, or Fe) with a similar water content (~50 wt%) were obtained through ion exchange by soaking Ca–Alg/D/Ca in a BaCl_2_, AlCl_3_, or FeCl_3_ solution, respectively, for one day (Fig. 3b). The swelling ratio of Ca–Alg/D/Ca–Fe was 93% (Supplementary Fig. 12). Energy-dispersive X-ray spectroscopy (EDX) mapping of the cross-section and X-ray photoelectron spectroscopy (XPS) analysis of the upper surface of Ca–Alg/D/Ca–Fe confirmed that secondary crosslinking ions (Fe^3+^) were homogeneously distributed throughout the hydrogel and completely replaced the pre-existing Ca^2+^ ions in the hydrogel (Fig. 3c,d). Consequently, the resulting reconstructed hydrogel possessed broad ranges of exceptional tensile strengths (8–57 MPa) and elastic moduli (94–1,290 MPa) (Fig. 3e, f). Crosslinking ions with a stronger binding affinity with Alg, in the order of Ca^2+^ < Ba^2+^ < Al^3+^ < Fe^3+^, formed stronger Alg networks, which results in higher tensile strength and elastic modulus. Notably, the tensile strength (57 MPa) and elastic modulus (1290 MPa) of Ca–Alg/D/Ca–Fe reached remarkably high values that have not been previously achieved in a wet polymeric hydrogel material, to the best of our knowledge. The mechanical performance of this hydrogel was also well maintained soaking in pure water (Supplementary Fig. 13).

Because producing thin hydrogels with mechanical stability and consistent performance is important for the practical use of hydrogels as a flexible backbone of bioelectronics and a solid electrolyte membrane, very thin Ca–Alg/D/Ca–Fe hydrogels with a thickness of 60–170 µm were fabricated and subjected to tensile tests (Fig. 3g). The thin hydrogels still exhibited good mechanical performance. In addition, because this hydrogel is composed of biopolymer alginate (extracted from sea brown algae) and exhibits almost no cytotoxicity (Supplementary Fig. 14), it could be utilized as a bio-friendly material.

Next, we compared the reconstruction method (anisotropic drying/shrinkage of pre-gel attached on the flat substrate and subsequent crosslinking/rehydration) and the conventional drawing method. The drawing process typically includes a drying/shrinkage of material fixed both ends (stretched condition) to align polymer chains along the longitudinal (stress) direction (Supplementary Fig. 15a)^30,42^. Although the resulting hydrogel exhibited enhanced mechanical properties compared to the initial hydrogel, it did not reach the strength and stiffness of the reconstructed Ca–Alg/D/Ca hydrogel (Supplementary Fig. 15b). The effect of Alg densification and Alg concentration increase in the hydrogel was larger in the reconstruction method than in the drawing method (Supplementary Fig. 15c), which yielded more strengthened and stiffened hydrogel (Ca–Alg/D/Ca). Moreover, after the ion exchange from Ca^2+^ to Fe^3+^, while the Ca–Alg/D/Ca–Fe hydrogel exhibited stably enhanced mechanical performance, the hydrogel obtained from the drawing method was brittle (Supplementary Fig. 15d).

### Mechanically robust and conductive hydrogels

**Fig. 4:**
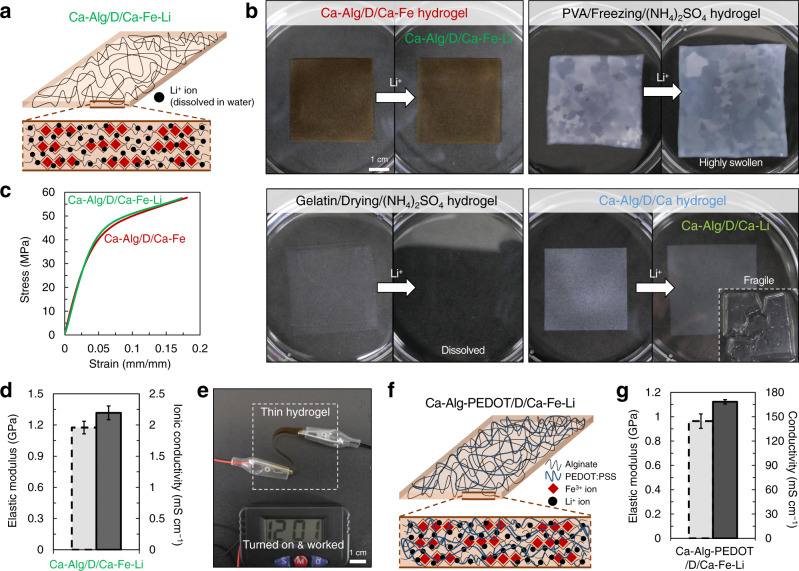
Mechanically robust, conductive hydrogels applicable as a gel electrolyte. **a** Illustration of Ca–Alg/D/Ca–Fe–Li hydrogel for electrolyte. **b** Photograph showing the fabrication of Ca–Alg/D/Ca–Fe–Li hydrogel and its stability in Li^+^ solution, and low mechanical stability of other hydrogels (PVA, gelatin, and Ca–Alg/D/Ca) in Li^+^ solution. **c** Stress–strain curves for Ca–Alg/D/Ca–Fe hydrogel before and after being soaked in Li^+^ solution. **d** Elastic modulus and ionic conductivity of Ca–Alg/D/Ca–Fe–Li hydrogel. **e** Photograph showing stable working performance of the Ca–Alg/D/Ca–Fe–Li hydrogel as a solid gel electrolyte. **f** Illustration of PEDOT-contained reconstructed hydrogel, Ca–Alg-PEDOT/D/Ca–Fe–Li. **g** Elastic modulus and conductivity of Ca–Alg-PEDOT/D/Ca–Fe–Li hydrogel. Error bars correspond to standard deviations.

We next evaluated the potential usability of superstrong and superstiff Ca–Alg/D/Ca–Fe hydrogel as a solid gel electrolyte membrane for energy storage devices. Ca–Alg/D/Ca–Fe was fully soaked in a 1 M Li^+^ aqueous solution to load Li^+^ ions within the hydrogel matrix (Ca–Alg/D/Ca–Fe–Li) (Fig. 4a, b); 1 M is a typical concentration of commercial liquid electrolytes^17^. For comparison, other biocompatible hydrogels with high mechanical properties, including polyvinyl alcohol (PVA), gelatin, and Ca–Alg/D/Ca hydrogels, were immersed in the Li^+^ solution (Fig. 4b). Although the PVA and gelatin hydrogels were fabricated by a method (using the Hofmeister effect/salting-out effect) that was recently suggested for producing hydrogels with superior mechanical properties^43,44^, these hydrogels were significantly swollen and weakened (PVA) or finally dissolved (gelatin) in the Li^+^ solution due to the salting-in effect^43^. The Ca–Alg/D/Ca hydrogel also became swollen and fragile in the Li^+^ solution because the relatively weaker Ca-crosslinks were dissociated by the excess Li^+^ ions; the Ca–Alg/D/Ca hydrogel soaked in the Li^+^ solution (Ca–Alg/D/Ca–Li) was significantly weakened (Supplementary Fig. 16). The wet glass fiber membrane, widely used as a separator for aqueous energy storage devices, was also significantly weak (Supplementary Fig. 17). In contrast, the Ca–Alg/D/Ca–Fe hydrogel retained its integrity in Li^+^ solutions without a notable change in its morphology and dimensions (Fig. 4b, Supplementary Fig. 18). The significantly densified Alg networks with strong Fe^3+^-crosslinks were stable, which led to the preservation of the original mechanical performance of Ca–Alg/D/Ca–Fe in the Li^+^ solution (Fig. 4c,d). Consequentially, the tensile strength (55 MPa) and elastic modulus (1.2 GPa) of Ca–Alg/D/Ca–Fe–Li (fully wetted within the Li^+^ solution) are comparable to or even superior to those of several conventionally used separators made of synthetic polymers^5,17,18,45,46^.

Ca–Alg/D/Ca–Fe–Li exhibited a high ionic conductivity of ~2.2 mS cm^−1^ (Fig. 4d), which is comparable to that of the conventional system consisting of liquid electrolyte and separator (1–4 mS cm^−1^)^17,47^. Since Ca–Alg/D/Ca–Fe contained sufficient water, the free Li^+^ ions dissolved in the water within the gel were mobile throughout the hydrogel. When the ions were removed from the hydrogel in pure water, the hydrogel exhibited no ionic conductivity (Supplementary Fig. 19), which clearly indicates that the ionic conductivity was attributed to the mobile Li^+^ ions within the hydrogel. The resultant hydrogel with mechanical robustness and good ionic conductivity worked well as a solid electrolyte membrane even in the bent state (Fig. 4e). Depending on the purpose of the electrolyte membrane, the elastic modulus (0.46 GPa) and ionic conductivity (7.8 mS cm^−1^) of hydrogel could be additionally controlled by using different Alg-crosslinking ions, Al^3+^ (Supplementary Fig. 20).

The proposed reconstruction method can be extended for fabricating a mechanically robust, electrically conductive hydrogel. For securing electrical conductivity, we chose the most well-known electrically conducting polymer, PEDOT:PSS, which is also biocompatible like Alg^48^. Because polyanionic Alg chains can partially replace anionic PSS chains from PEDOT:PSS (binding energy: PEDOT–Alg > PEDOT–PSS)^49^, the commercial PEDOT:PSS dispersion, a polyelectrolyte complex of cationic PEDOT and anionic PSS, was well dispersed in Alg solution over mixing. Through the reconstructing process, we easily obtained a conductive Ca–Alg-PEDOT/D/Ca–Fe hydrogel (Fig. 4f). The electrical conductivity of the resulting hydrogel was first identified by comparing Ca–Alg-PEDOT/D/Ca–Fe-Water (119.4 mS cm^−1^) and Ca–Alg/D/Ca–Fe-Water (0.0 mS cm^−1^) that does not contain Li^+^ ions (Supplementary Table 1). Meanwhile, the PEDOT-contained hydrogel with Li^+^ ions (Ca–Alg-PEDOT/D/Ca–Fe–Li) exhibited high conductivity of 168.4 mS cm^−1^ that is superior to that of Ca–Alg/D/Ca–Fe–Li (2.2 mS cm^−1^), due to the combination of mobile ions and electrically conducting polymer. The elastic modulus of Ca–Alg-PEDOT/D/Ca–Fe–Li was still high as approximately 1.0 GPa (Fig. 4g).

### Aqueous supercapacitor

**Fig. 5:**
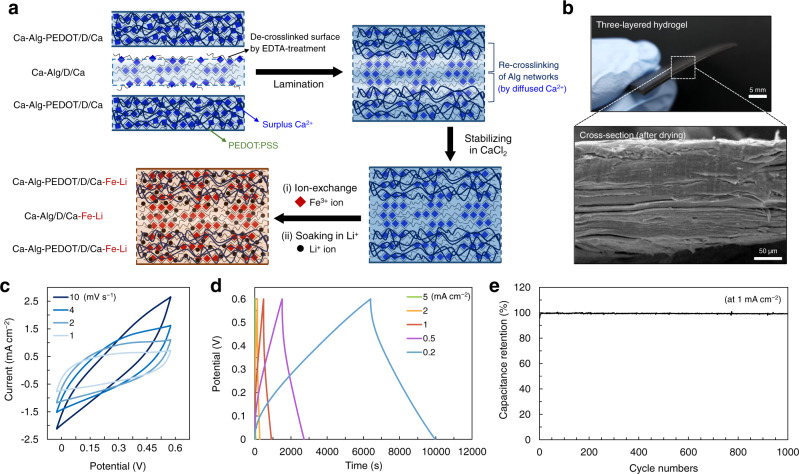
Aqueous supercapacitor composed of AC electrodes and three-layered hydrogel electrolyte. **a** Schematic of fabricating three-layered hydrogel electrolyte. **b** Photograph and cross-sectional SEM image of the three-layered hydrogel. **c** Cyclic voltammetry (CV) curves at different scan rates. **d** Galvanostatic charge-discharge (GCD) curves at different current densities. **e** Capacitance retention during 1000 charge-discharge cycles at a current density of 1 mA cm^−2^, showing stable cycle performance.

A supercapacitor cell was prepared to investigate the potential and practical usability of the above-developed electrical- and/or ionic-conductive hydrogels (Fig. 1d). For an electrolyte, we fabricated a three-layered laminated hydrogel composed of two conductivity-improved PEDOT-contained hydrogel layers (Ca–Alg–PEDOT/D/Ca–Fe–Li) and an intermediate ionic-conductive hydrogel layer (Ca–Alg/D/Ca–Fe–Li) (Fig. 5a, Supplementary Fig. 7). The laminated hydrogel of three layers was stable without distinct borders (Fig. 5b). As an electrode, activated carbon, the most commonly used electrode material for supercapacitors, was used.

The supercapacitor electrochemical performance was analyzed by cyclic voltammetry (CV) and galvanostatic charge-discharge (GCD) tests. The device exhibited typical supercapacitor CV curves with symmetrical shapes at scan rates of 1–10 mV s^−1^ (Fig. 5c). GCD curves were also typical triangular shapes at current densities of 0.2–5 mA cm^−1^ (Fig. 5d). The supercapacitor composed of single-layer hydrogel electrolyte without PEDOT (Ca–Alg/D/Ca–Fe–Li) exhibited less capacitance (Supplementary Fig. 21), which implies that the three-layered electrolyte membrane with outer PEDOT-contained layers could be better for energy storage devices. (A detailed study about the supercapacitor performance depending on the difference between the single-layer and three-layered electrolyte membranes will be discussed elsewhere in the near future.) Further, this device exhibited superior electrochemical stability; during over 1000 charge/discharge cycles for 12 days, there was little reduction in capacitance retention (almost 100 % retention) (Fig. 5e). These data represent that the reconstructed hydrogels with high and stable mechanical and conductive performances have the potential for practical application as a solid gel electrolyte membrane.

In summary, we have developed mechanically enhanced hydrogels based on a simple reconstruction method yielding densely interconnected polymer networks. The anisotropic drying/shrinkage densified polymer chains, and the subsequent ionic crosslinking in an ionic solution fixed the compact network and led to rehydration. Such facile processes were available for large-scale production of hydrogels, which are expected to be adaptable for practical manufacturing. The resulting reconstructed hydrogel possessed exceptional tensile strengths and elastic moduli in the broad ranges of 8–57 MPa and 94–1,290 MPa, respectively, depending on the crosslinking ions. Furthermore, through the addition of the conducting polymer PEDOT, the ionic-/electrical-conductive hydrogel was producible. In addition to the mechanical performance, because the hydrogel conductivity was sufficiently high for practical application as solid gel electrolyte membranes^5,18,46,50^, we demonstrated the practical usability of the hydrogel as the gel electrolyte membrane for a supercapacitor. Based on this report, we expect to further develop mechanically robust functional hydrogels by combining the present concept with existing approaches, for example, adding other polymers^51^, drawing polymer chains^52,53^, or embedding inorganic particles^6,54^.

## Methods

### Materials

Alginic acid sodium salt from brown algae (Alg, medium viscosity), poly(vinyl alcohol) (PVA, M_w_ = 130,000, 99+% hydrolyzed), gelatin from porcine skin (Type A), poly(3,4-ethylenedioxythio phene):poly(styrene sulfonate) (PEDOT:PSS, 1.3 wt% dispersion in H_2_O, conductive grade, 1 S/cm), polytetrafluoroethylene (PTFE), calcium chloride dihydrate (CaCl_2_·2H_2_O, assay ≥ 99.0%), barium chloride dihydrate (BaCl_2_·2H_2_O, assay ≥ 99%), aluminum chloride hexahydrate (AlCl_3_·6H_2_O, assay ≈ 99%), iron chloride (FeCl_3_, assay ≈ 97%), and lithium sulfate (Li_2_SO_4_, assay ≥98.5% (titration)) were purchased from Sigma-Aldrich. Calcium sulfate dihydrate (CaSO_4_·2H_2_O, assay ≥ 98.0%), lithium bromide anhydrous (LiBr, assay ≥ 99.0%), ammonium sulfate ((NH_4_)_2_SO_4_, assay ≥ 99.0%), and ethylenediaminetetraacetic acid (EDTA, assay ≥ 99.0%) were purchased from Samchun (South Korea). The glass fiber membrane was purchased from Whatman, Cytiva (USA). The activated carbon (surface area = 1900–2200 m^2^/g) was purchased from Power Carbon Technology (South Korea). The materials were used as received without further purification. Dulbecco’s modified Eagle medium (DMEM) with high glucose and sodium pyruvate was purchased from Gibco. Fetal bovine serum (FBS), penicillin/streptomycin, and cell counting kit-8 (CCK-8) were purchased from Merck (Germany), Lonza (Switzerland), and Dojindo (Japan), respectively.

### Preparation of reconstructed hydrogels

Alginic acid sodium salt (Alg) was dissolved in distilled water and mixed with a calcium sulfate slurry (CaSO_4_) to obtain a 2 wt/vol(%) Alg mixture. The amount of CaSO_4_ added to the Alg solution was 15 wt% to Alg (Ca^2+^ ion/Alg-COO^–^ ratio = 15.3 mol%). The mixture was immediately molded and gelated between glass plates with 3 mm thick spacers. This mixture was stored in a 5 °C cooler for 1 day to complete ionic crosslinking. The prepared pre-gel (Ca–Alg) was anisotropically dried/shrunk on a flat dish at 20–25 °C and 20–25% RH for 3 days. The obtained dried sheets (Ca–Alg/D) were soaked in an excess of 100 mM CaCl_2_ solution for 1 day. Finally, the rehydrated hydrogels were rinsed using distilled water and soaked in an excess of 200 mM CaCl_2_, BaCl_2_, AlCl_3_, or FeCl_3_ for 1 day (Ca–Alg/D/Ca or Ca–Alg/D/Ca–M). In the case of electrically conductive Ca–Alg-PEDOT/D/Ca–Fe hydrogel, a mixture solution of Alg and PEDOT:PSS (Alg/PEDOT:PSS wt ratio = 1/0.72) was first prepared, and then fabricated by the same way above-described. For a thick bulky hydrogel, the Ca–Alg/D/Ca hydrogels were laminated. The surface of Ca–Alg/D/Ca hydrogels was first treated with a 1 M EDTA solution, and stacked and pressed for re-crosslinking between the adjacent hydrogels. Then, the laminated hydrogel was stored in CaCl_2_ to completely form and stabilize the interfacial bonding.

### Preparation of Li^+^-loaded hydrogels

The PVA, gelatin, Ca–Alg/D/Ca, Ca–Alg/D/Ca–Fe, and Ca–Alg-PEDOT/D/Ca–Fe hydrogels were soaked in a 1 M LiBr aqueous solution for 3 days. The Li^+^ concentration was referred to typical liquid electrolytes, e.g., 1 M LiTFSI, 1 M LiBF_4_, or 1 M LiPF_6_ dissolved in organic solvents^17,45,55–57^. For mechanically robust PVA and gelatin hydrogels preparation, the salting-out effect was employed^43,44^. A 5 wt% PVA solution was prepared by dissolving PVA powder in distilled water at 90–95 °C. The PVA solution was poured into Petri dish and frozen at −20 °C. Then, a 1 M (NH_4_)_2_SO_4_ solution was added to the frozen sample at 20–25 °C. After 1 day, a PVA/Freezing/(NH_4_)_2_SO_4_ hydrogel was obtained. For a gelatin hydrogel, a 5 wt% gelatin solution was prepared by dissolving gelatin powder in distilled water at 50 °C. The gelatin solution was poured into Petri dish, stored at 5 °C for 1 day, and then dried at 20–25 °C. Next, a 1 M (NH_4_)_2_SO_4_ solution was added to the dried sample to obtain a gelatin/drying/(NH_4_)_2_SO_4_ hydrogel.

### Preparation of aqueous supercapacitor

The three-layered electrolyte (Ca–Alg-PEDOT/D/Ca–Fe–Li, Ca–Alg/D/Ca–Fe–Li, and Ca–Alg-PEDOT/D/Ca–Fe–Li) was prepared as follows. The Ca–Alg/D/Ca hydrogel surface was treated with 1 M EDTA solution and staked in between the Ca–Alg-PEDOT/D/Ca hydrogels under gentle pressure for re-crosslinking between the adjacent hydrogels. The laminated hydrogel was then soaked in a 200 mM CaCl_2_ solution for a few hours, followed by ion exchange in a 200 mM FeCl_3_ solution for a day. The final reconstructed hydrogel was then washed with a 1 M Li_2_SO_4_ several times and stored in the Li_2_SO_4_ solution to obtain Li-loaded electrolyte membranes. In the electrode case, a slurry composed of activated carbon and PTFE (9:1 ratio) with a little ethanol was blade-casted and dried to obtain an electrode of 300 µm thickness. Then, the wet electrolyte membrane and electrodes were packaged into a 2032 coin cell.

### Mechanical tests

Mechanical tests were conducted using a Comtech QC-508E universal testing machine with a load speed of 10 mm/min using a 100 N or 500 N load cell. Rectangular specimens (10 mm width and 40–50 mm length) were prepared for tensile testing (*n* > 5). The thickness of hydrogels was measured carefully using a microcaliper. The gauge length between the grips was approximately 15 mm.

### In vitro cytotoxicity test

For evaluating the cytotoxicity of Ca–Alg/D/Ca–Fe, the viability of L6 myoblast cells with and without the hydrogel was compared. The L6 myoblast cells (1 × 10^4^) were first seeded in a 6-well plate with 1 mL DMEM supplemented with 10 % FBS and 1% penicillin/streptomycin. After 18 h, the hydrogel of 2 mg, 10 mg, or 20 mg (*n* = 4) was added to the Transwell and incubated within the cell-cultured plate at 37 °C in 5% CO_2_ for 24 h. Before being added to the Transwell, the hydrogels were washed with pure water and DMEM solution and sterilized under UV light for a few hours. The cell viability was measured using the CCK-8 assay, according to the manufacturer’s instructions.

### Characterization

The water content of the hydrogel was calculated from the weight change before/after drying under vacuum for 2 days. Scanning electron microscopy (SEM) images were obtained using a JEOL JSM7500F or JSM-6510 instrument. Energy-dispersive X-ray spectroscopy (EDX) mapping was performed using a JEOL JSM7500F instrument. Surface hardness was analyzed using a nanoindenter (Micro Materials NanoTest Vantage Platform). The surface hardness value is an average of random ten points at 1.2 µm depth of each sample. X-ray diffraction (XRD) analysis was performed under ambient conditions in air using at a scanning speed of 3° min^−1^ (Bruker Corporation D8 ADVANCE). X-ray photoelectron spectroscopy (XPS) was performed using a Thermo Scientific ESCALAB 250 instrument. Fourier-transform infrared (FTIR) spectra data were obtained using an ATR (attenuated total reflectance) mode of Bruker IFS-66/S, TENSOR27 spectrometer. The flat hydrogel sample was placed on a ZnSe crystal and analyzed. Differential scanning calorimetry (DSC) analysis was performed using a HITACHI DSC6100 instrument under nitrogen atmosphere. The sample was first fully rinsed with pure water to completely remove unreacted residual ions, and then cooled from room temperature to −60 °C and then heated to 50 °C at a rate of 3 °C min^−1^. To evaluate the ionic conductivity of hydrogels, the hydrogel was sandwiched between stainless steel electrodes and assembled into a 2032 coin cell. The resistance (R) of the hydrogel was obtained from electrochemical impedance spectroscopy (EIS) (VMP3, BioLogic) at room temperature. The resistance is the intercept on the real axis in the Nyquist plot at high frequencies, and the conductivity (σ) was calculated as *σ* = *t*/*R* · *A*, where t is the hydrogel thickness and A is the electrode area (1.767 cm^2^). The conductivity of PEDOT-contained hydrogel was measured through the four-point probe technique. The rectangular PEDOT-contained hydrogel (30 mm in length and 5 mm in width) was directly placed onto wire probes, sealed in Bekktech conductivity cell (BT-115), and its resistance (R) was measured using an impedance analyzer (Zive SP2, Wonatech) under a relative humidity of 100 % and 30 °C. The electrical conductivity was calculated as $\sigma =L/R\cdot W\cdot T$, where *L* is the distance between the two electrodes for voltage measurement (0.425 cm), *W* is the hydrogel width and *T* is the hydrogel thickness. The electrochemical performance of the supercapacitor was measured by electrochemical workstation (VSP, BioLogic) at room temperature.

### Reporting summary

Further information on research design is available in the Nature Research Reporting Summary linked to this article.

## Supplementary information

Supplementary Information

Reporting Summary

## Data Availability

The data that support the findings of this study are available from the corresponding author upon request.
