# Supplementary material for: Superstrong, superstiff, and conductive alginate hydrogels
Source: Nat Commun. 2022 May 31;13:3019. doi: 10.1038/s41467-022-30691-z (PMC9156673; doi:10.1038/s41467-022-30691-z)
Supplement: Supplementary file 1 — Supplementary Information [file 41467_2022_30691_MOESM1_ESM.pdf]

Supplementary Information

Superstrong, Superstiff, and Conductive Alginate Hydrogels

Donghwan Ji[1], *Jae Min Park*[1], *Myeong Seon Oh*[1], *Thanh Loc Nguyen*[1], *Hyunsu Shin*[1], *Jae Seong Kim*[1], *Dukjoon Kim*[1], *Ho Seok Park*[1], *Jaeyun Kim*[1,2,3,4]*

[1]School of Chemical Engineering, Sungkyunkwan University (SKKU), Suwon 16419, Republic of Korea

[2]Department of Health Sciences and Technology, Samsung Advanced Institute for Health Sciences & Technology (SAIHST), Sungkyunkwan University (SKKU), Suwon 16419, Republic of Korea

[3]Biomedical Institute for Convergence at SKKU (BICS), Sungkyunkwan University (SKKU), Suwon 16419, Republic of Korea

[4]Institute of Quantum Biophysics (IQB), Sungkyunkwan University (SKKU), Suwon 16419, Republic of Korea

* Correspondence should be addressed to Prof. J. Kim (E-mail: kimjaeyun@skku.edu).

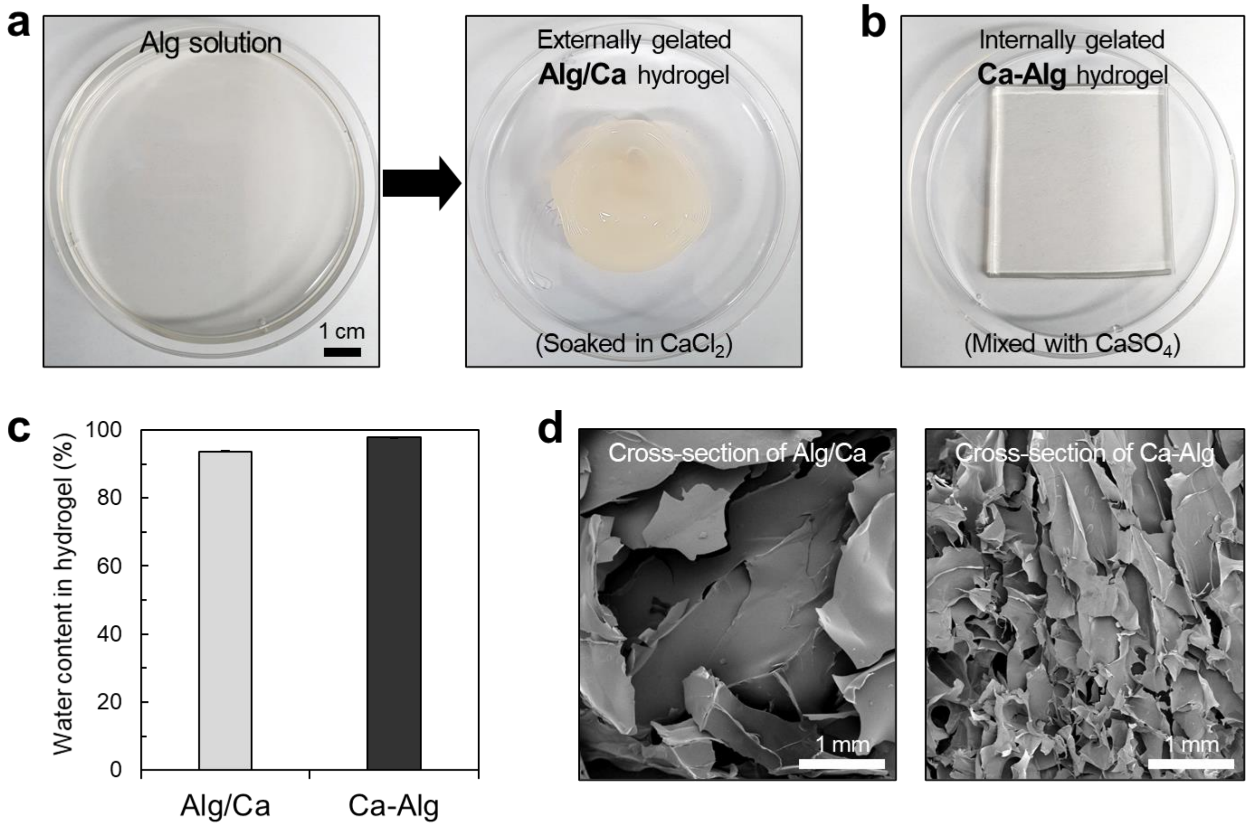

Supplementary Fig. 1. Alg hydrogels fabricated by conventional methods. (a, b) Photographs of Alg/Ca hydrogel externally gelated in $CaCl_2$ and Ca-Alg hydrogel internally gelated by $CaSO_4$ slurry. (c) Water content of each hydrogel. At least five samples were measured for the calculation of average values and standard deviations (error bars). (d) Cross-sectional SEM images of lyophilized hydrogels. Because both Alg/Ca and Ca-Alg hydrogels contain much water in sparse Alg networks, porous structures were observed.

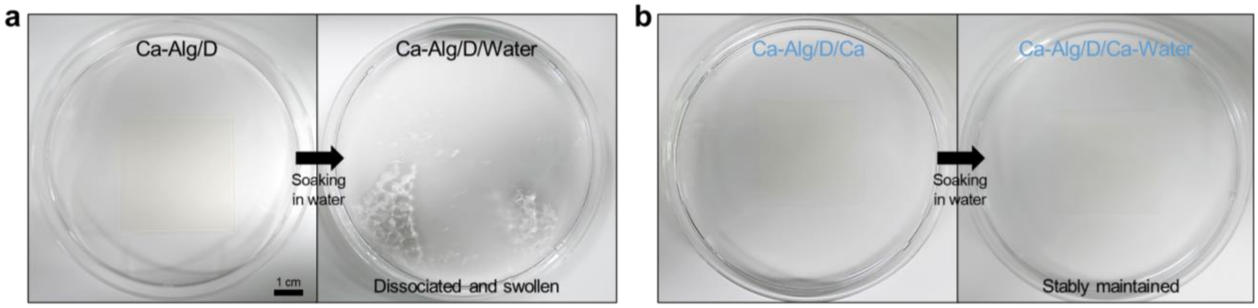

Supplementary Fig. 2. Comparison between weakly crosslinked Ca-Alg/D dried film and fully crosslinked Ca-Alg/D/Ca hydrogel. (a) Ca-Alg/D with the limited Ca^{2+} crosslinking was immediately swollen and completely dissociated in a few hours after being soaked in pure water. (b) Ca-Alg/D/Ca with dense and fully Ca^{2+} crosslinking was stably maintained for at least a week in pure water.

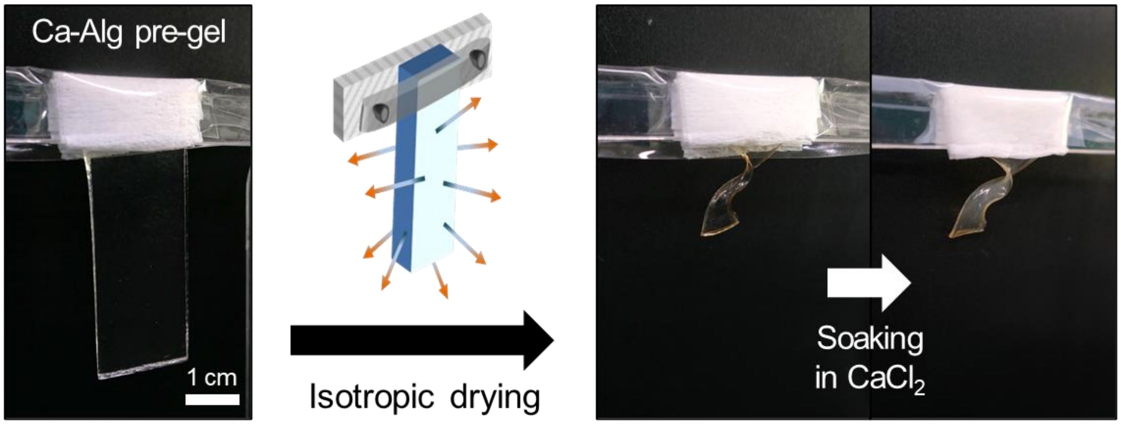

Supplementary Fig. 3. All-directional drying process producing warped hydrogel. For obtaining a flat hydrogel with uniformly densified polymer networks, the anisotropic drying/shrinkage process is crucial.

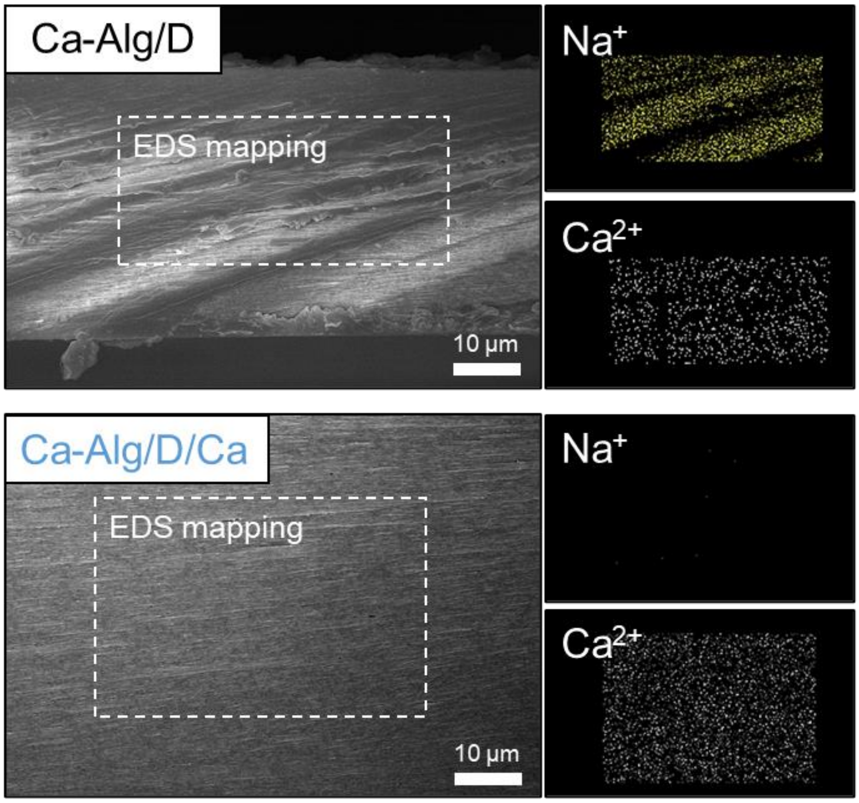

Supplementary Fig. 4. Effect of additional crosslinking/rehydration in Ca^{2+} solution. Before rehydration/crosslinking in $CaCl_2$, Ca-Alg/D contained many Na^+ ions because it was made from sodium alginic acid. However, the crosslinking/rehydration step completely removed the Na^+ ions and formed fully Ca-crosslinked Alg networks to produce the Ca-Alg/D/Ca hydrogel.

For 90 µm-thick hydrogel

Ca-Alg/D	
Dried film	
Element	wt%
C	37.7
O	51.0
Na	7.9
Ca	1.5
S	1.8
Cl	0.1
SUM	100.0

5 min →

Ca-Alg/D/Ca	
Soaked in $CaCl_2$ for 5 min	
Element	wt%
C	48.8
O	46.1
Na	0.1
Ca	4.3
S	0.6
Cl	0.2
SUM	100.0

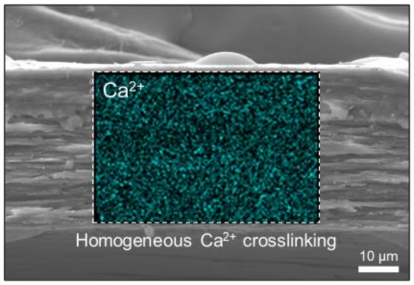

For 300 µm-thick hydrogel

Ca-Alg/D	
Dried film	
Element	wt%
C	38.9
O	51.7
Na	6.7
Ca	1.1
S	1.5
Cl	0.1
SUM	100.0

5 min →

Ca-Alg/D/Ca	
Soaked in $CaCl_2$ for 5 min	
Element	wt%
C	45.0
O	49.3
Na	2.8
Ca	2.0
S	0.5
Cl	0.5
SUM	100.0

10 min →

Ca-Alg/D/Ca	
Soaked in $CaCl_2$ for 15 min	
Element	wt%
C	46.0
O	47.8
Na	0.2
Ca	4.8
S	0.2
Cl	1.1
SUM	100.0

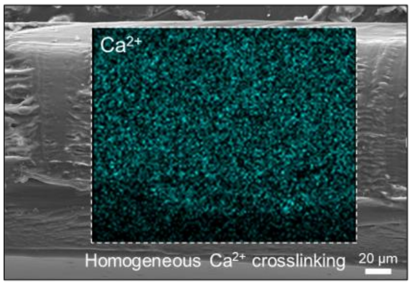

Supplementary Fig. 5. Homogenous Ca^{2+} crosslinking in Ca-Alg/D/Ca with different thicknesses. EDX mapping element analysis of cross-sectional Ca-Alg/D/Ca hydrogel with different thicknesses (e.g., 90 and 300 µm) demonstrated that the Ca^{2+} crosslinking was completed in 30 min and there was no inhomogeneity. The 90 µm-thick Ca-Alg/D/Ca hydrogel was obtained from the 3 mm-thick pre-gel, and the 300 µm-thick Ca-Alg/D/Ca hydrogel was obtained from the 10 mm-thick pre-gel.

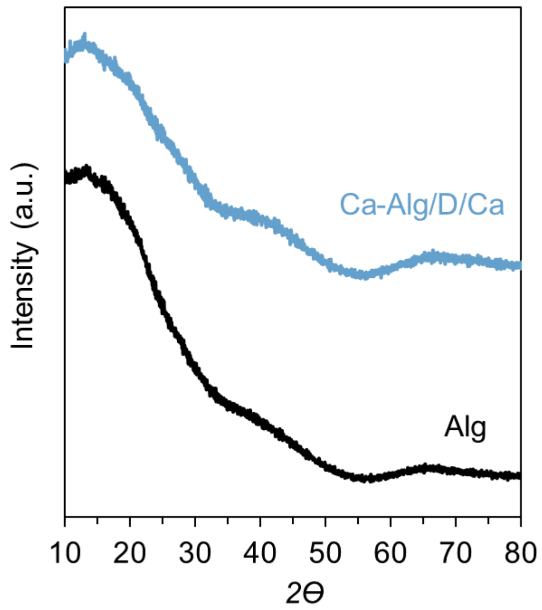

Supplementary Fig. 6. XRD patterns of before/after reconstructing process. There was no distinct difference in XRD pattern between Ca-Alg/D/Ca and Alg. Generally, Alg polymer is known as an amorphous polymer.

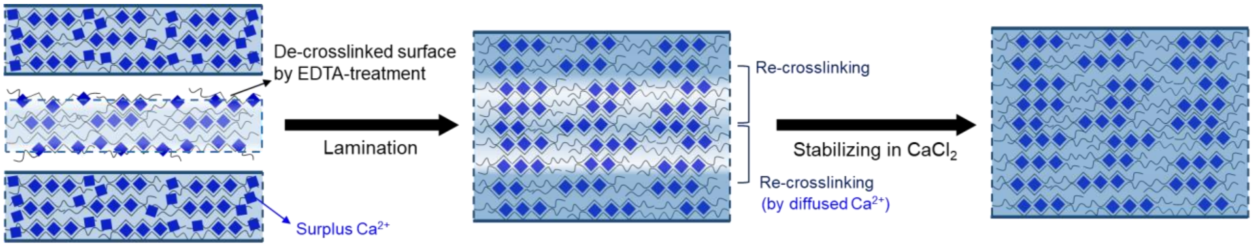

Supplementary Fig. 7. Lamination of Ca-Alg/D/Ca for thick bulky hydrogel. The thin Ca-Alg/D/Ca hydrogels were first laminated using ethylenediaminetetraacetic acid (EDTA) and then stored in $CaCl_2$ to completely form and stabilize the interfacial bonding.

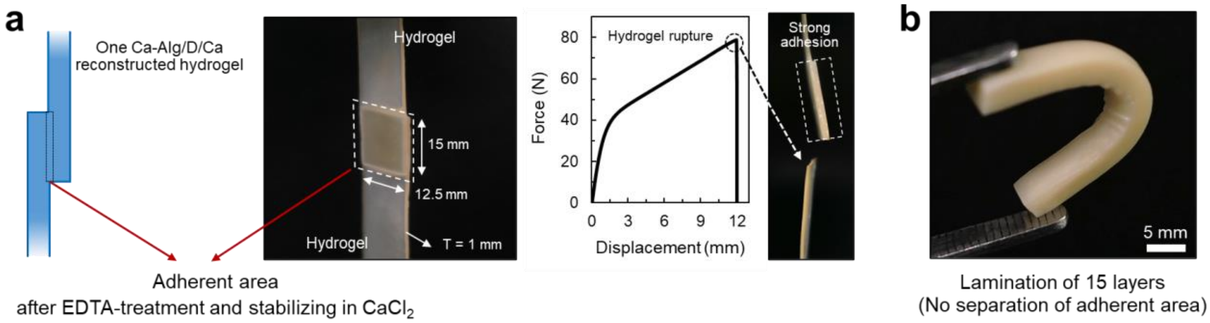

Supplementary Fig. 8. Strong and stable interfaces of laminated thick Ca-Alg/D/Ca hydrogel. (a) Lap-shear test demonstrates high adhesion strength between two reconstructed hydrogels. (b) Photograph exhibits the laminated hydrogel was bendable without interfacial separation due to strong and stable interfacial bonds.

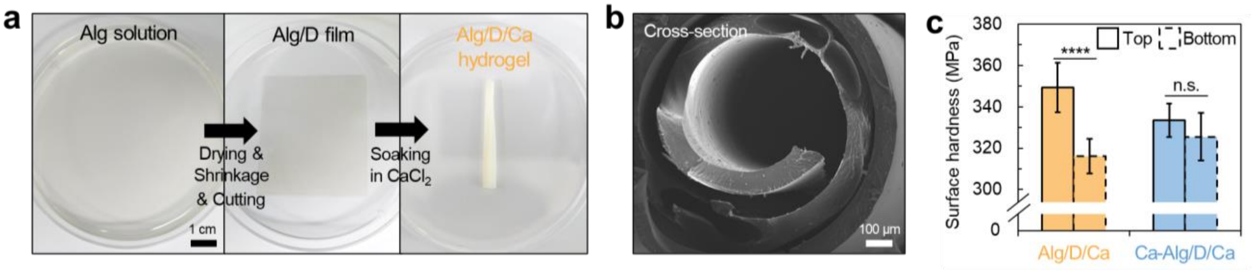

Supplementary Fig. 9. Non-uniform hydrogel obtained from Alg solution, not Ca-Alg pre-gel. (a) Photographs and (b) cross-sectional SEM image, showing mechanical instability of the hydrogel fabricated from Alg solution. (c) Difference in top and bottom surface hardness of dried Alg/D/Ca and Ca-Alg/D/Ca. The surface hardness value is an average of random 10 points at 1.2 µm depth of each sample and error bars correspond to standard deviations. P values were determined by a Student's t-test (****$P < 0.0001$; n.s., not significant), and the statistical significance (P value) was the same regardless of one-sided and two-sided tests.

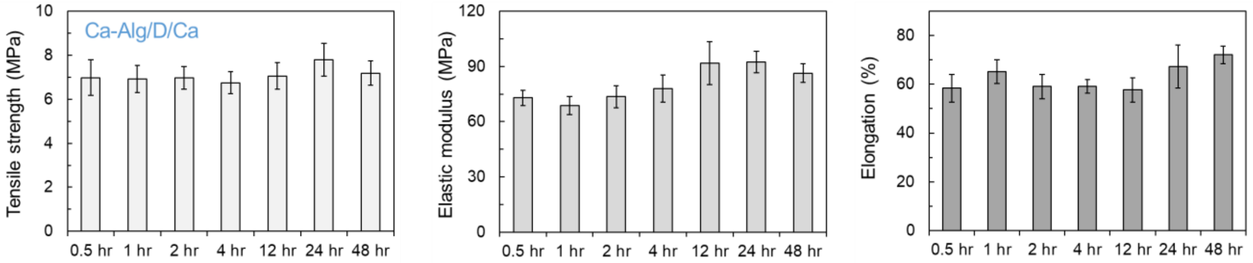

Supplementary Fig. 10. Effect of $CaCl_2$ soaking time on mechanical properties of Ca-Alg/D/Ca. The crosslinking/rehydration of the dried Ca-Alg/D film was completed in a short time. As a result, the produced Ca-Alg/D/Ca hydrogel exhibited no significant difference in the mechanical properties with variation of the soaking time. Error bars correspond to standard deviations.

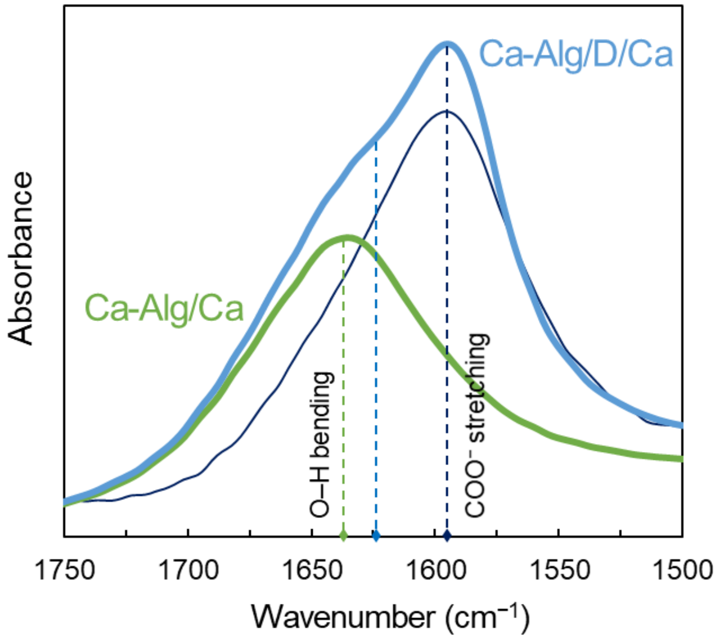

Supplementary Fig. 11. FTIR spectra of Ca-Alg and Ca-Alg/D/Ca hydrogels. The strongest O–H bending peak intensity in Ca-Alg/Ca was at the wavenumber of 1635 cm^{-1}, whereas the strongest O–H bending peak intensity in Ca-Alg/D/Ca was at 1625–1635 cm^{-1}.

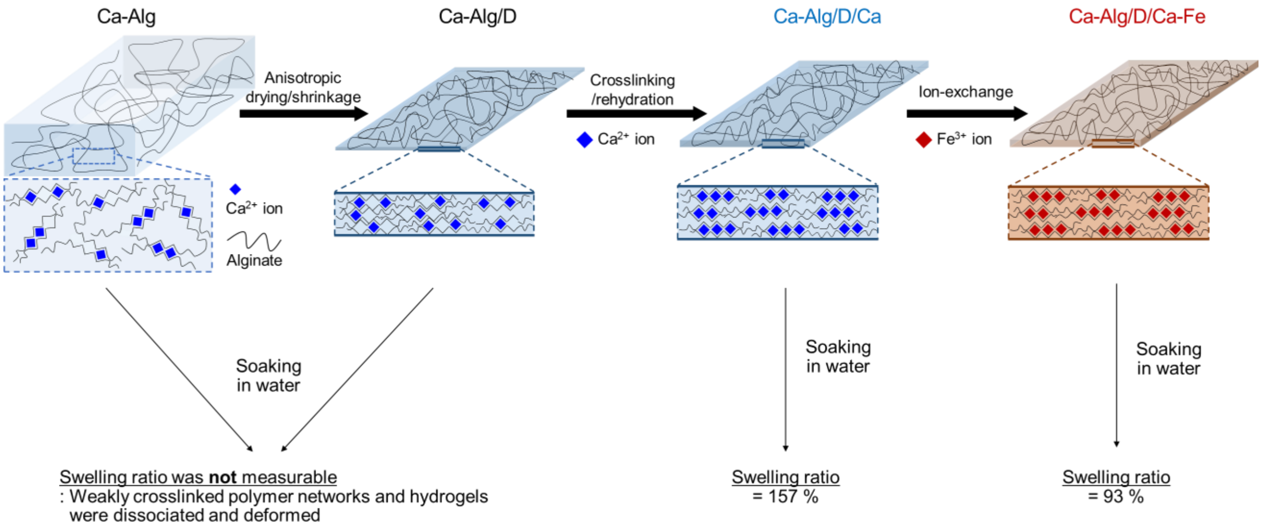

Supplementary Fig. 12. Swelling ratio of the reconstructed hydrogels, Ca-Alg/D/Ca and Ca-Alg/D/Ca-Fe. Because the Ca-Alg pre-gel and the Ca-Alg/D dried film were weakly crosslinked, they were completely dissociated and deformed in pure water (Supplementary Fig. 2a). In contrast, the reconstructed hydrogels were stable in pure water (Supplementary Fig. 2b). Since Fe^{3+} crosslinking for Alg networks is stronger than Ca^{2+} crosslinking, the swelling ratio became low in the Ca-Alg/D/Ca-Fe hydrogel. The swelling ratio of Ca-Alg/D/Ca-Fe was over 90 % in pure water, and its mechanical properties were almost consistent regardless of the water-soaking step (See Supplementary Fig. 13). The swelling ratio was calculated as follows:

$$swelling\ ratio = \frac{Weight_{swollen\ hydrogel} - Weight_{dried\ sample}}{Weight_{dried\ sample}} \times 100\ (\%).$$

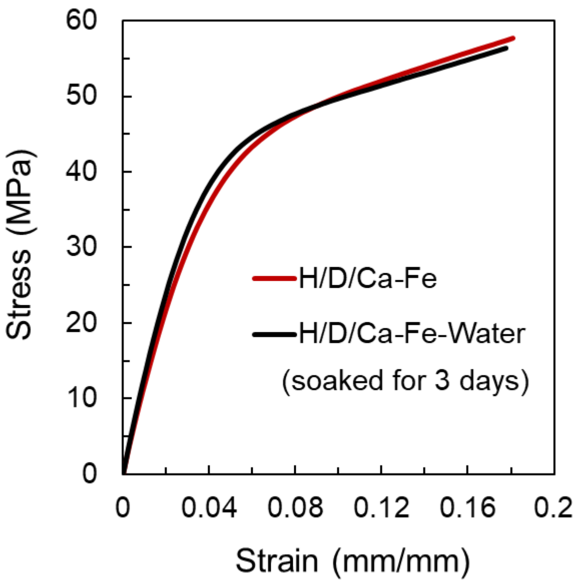

Supplementary Fig. 13. Mechanical stability of Ca-Alg/D/Ca-Fe hydrogel. The strong and stiff Ca-Alg/D/Ca-Fe hydrogel exhibited consistent mechanical performance after being soaked in pure water for a period of 3 days. (Ca-Alg was marked as H in stress-strain curves. H is an acronym of a hydrogel.)

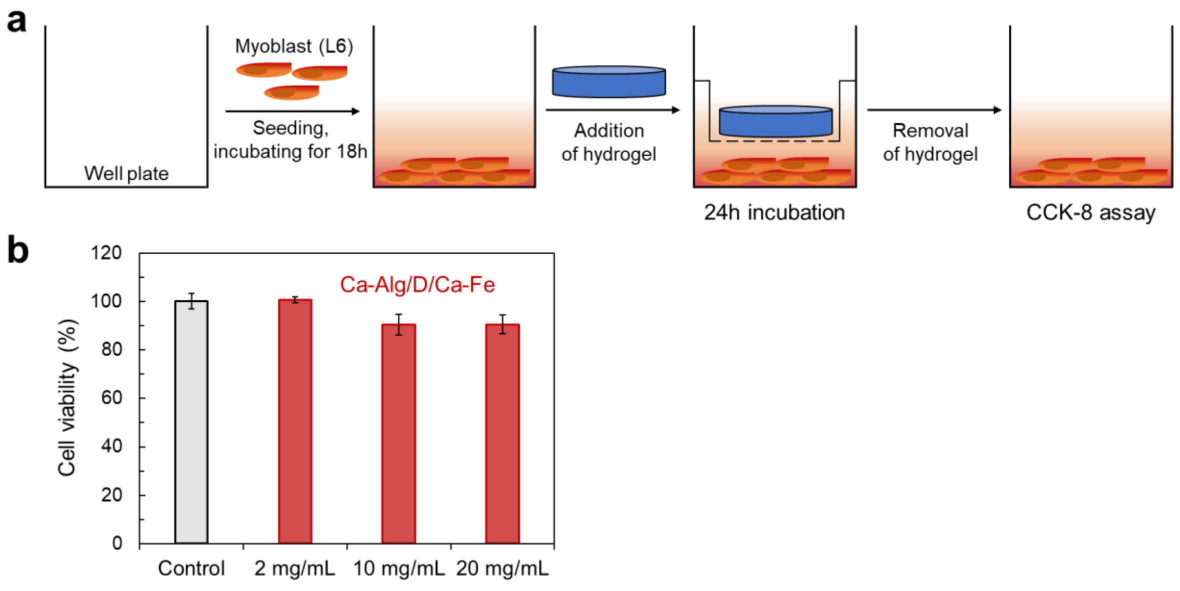

Supplementary Fig. 14. In vitro cell cytotoxicity of Ca-Alg/D/Ca-Fe. (a) Schematic of in vitro cell cytotoxicity test. (b) Cell viability measured using the cell counting kit-8 (CCK-8) assay. The strong and stiff Ca-Alg/D/Ca-Fe hydrogel of 2 mg, 10 mg, and 20 mg (n=4) was co-cultured within the 1 mL DMEM of 4×10^4 L6 myoblast cells and demonstrated no cytotoxicity. Error bars correspond to standard deviations.

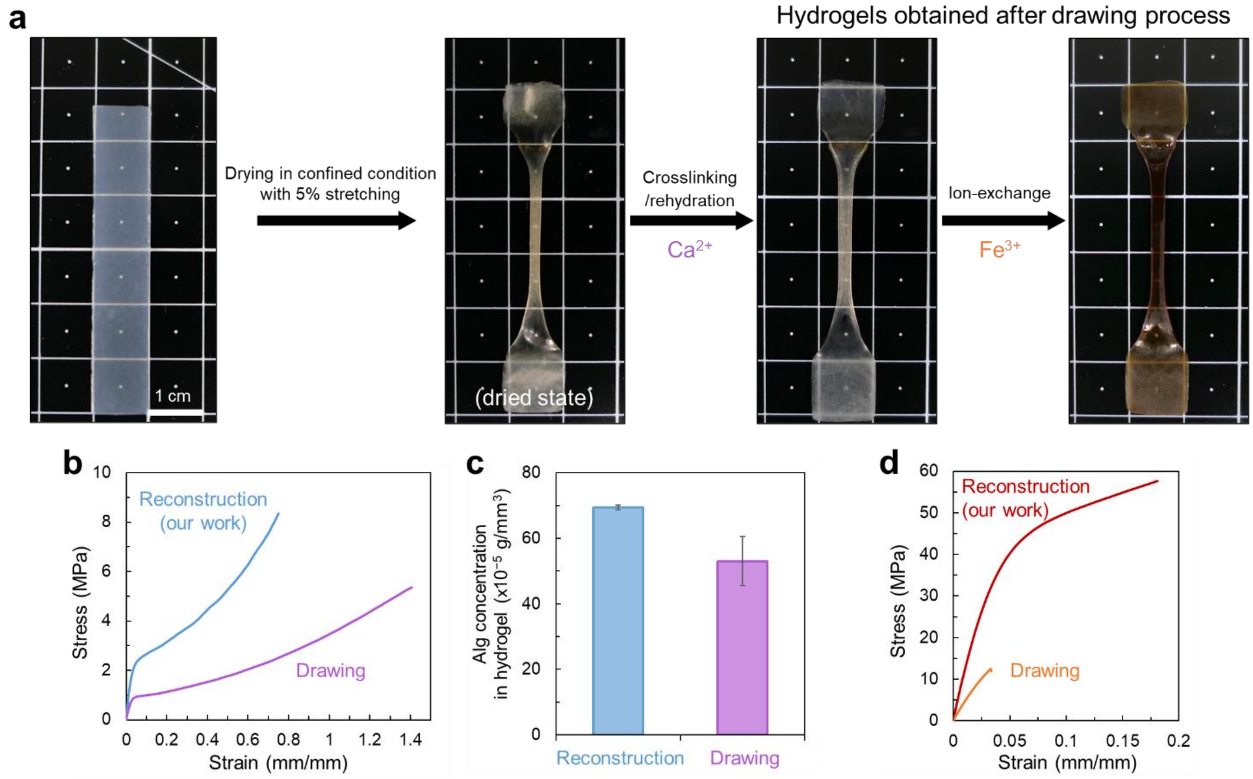

Supplementary Fig. 15. Mechanically reinforced hydrogel through drawing method. (a) Photographs showing the hydrogel sample at each step. The drawing method is composed of drying in confined condition with some stretching and crosslinking/rehydration in an ionic solution. (b) Stress-strain curve of a hydrogel fabricated through the reconstruction method (blue) and a hydrogel fabricated through the drawing method (purple). (c) Alginate concentration in each hydrogel. Error bars correspond to standard deviations. (d) Stress-strain curve of each hydrogel after ionic exchange from Ca^{2+} to Fe^{3+} in $FeCl_3$ solution.

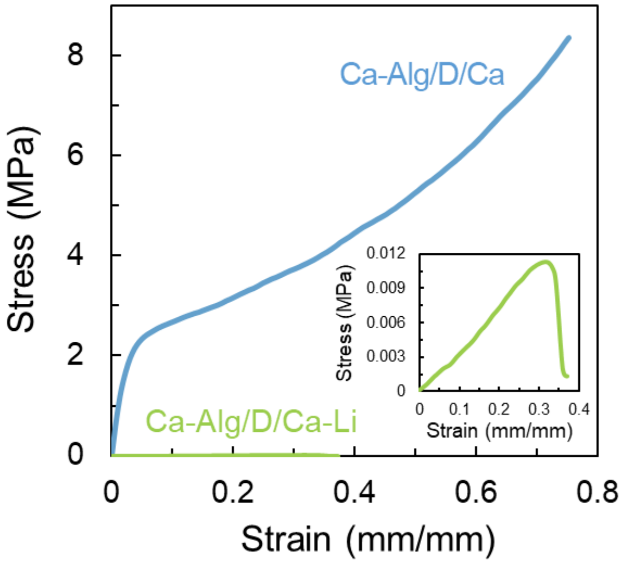

Supplementary Fig. 16. Stress-strain curves for Ca-Alg/D/Ca before and after soaking in Li^+ aqeuous solution. The Ca-Alg/D/Ca hydrogel is significantly weakened in the Li^+ solution.

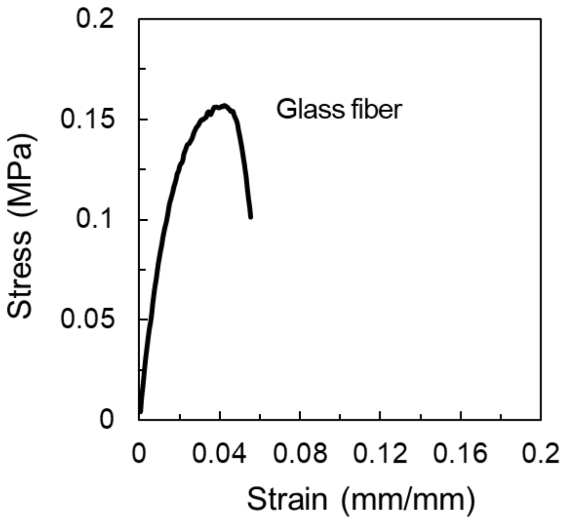

Supplementary Fig. 17. Stress strain curves of glass fiber membrane wetted in pure water.

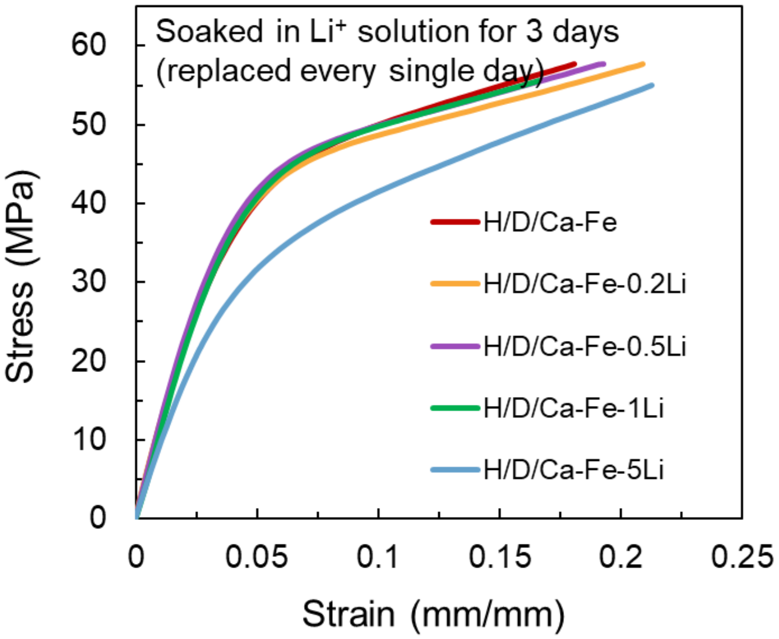

Supplementary Fig. 18. Mechanical properties of Ca-Alg/D/Ca-Fe hydrogel before and after soaked in Li^+ solution with different concentrations. While the Ca-Alg/D/Ca-Fe hydrogels were being soaked in 0.2, 0.5, or 1 M Li^+ solution for 3 days, there was no notable change in the hydrogel mechanical performance. Although the hydrogel soaked in 5 M Li^+ solution was slightly weakened, the fracture strength was still maintained. (Ca-Alg was marked as H in stress-strain curves. H is an acronym of a hydrogel.)

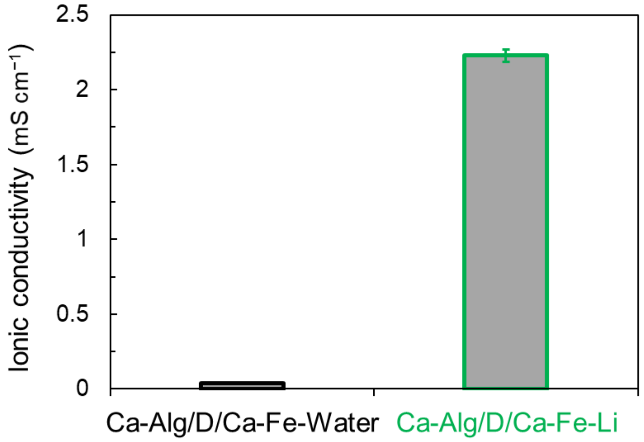

Supplementary Fig. 19. Ionic conductivity of Ca-Alg/D/Ca-Fe after soaking in pure water or Li^+ aqueous solution. When the mobile ions were not contained in the hydrogel, the hydrogel exhibited no conductivity.

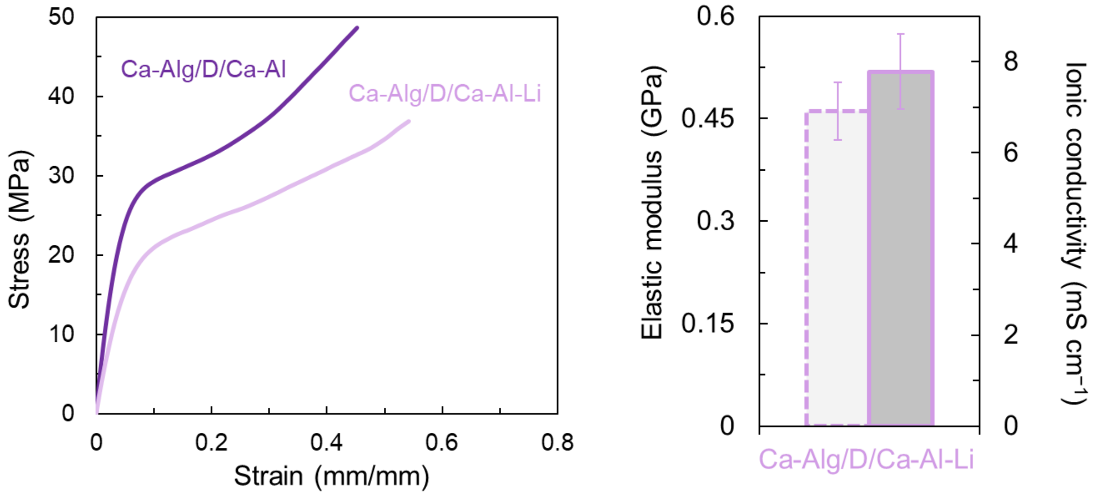

Supplementary Fig. 20. Stress-strain curves of Ca-Alg/D/Ca-Al before/after soaking in Li^+ aqueous solution. Because the strength of Al^{3+} crosslinking is weaker than that of Fe^{3+} crosslinking, Ca-Alg/D/Ca-Al was slightly swollen the in Li^+ solution, resulting in Ca-Alg/D/Ca-Al-Li possessed higher ionic conductivity with more sparse polymer networks than that of Ca-Alg/D/Ca-Fe-Li. Error bars correspond to standard deviations.

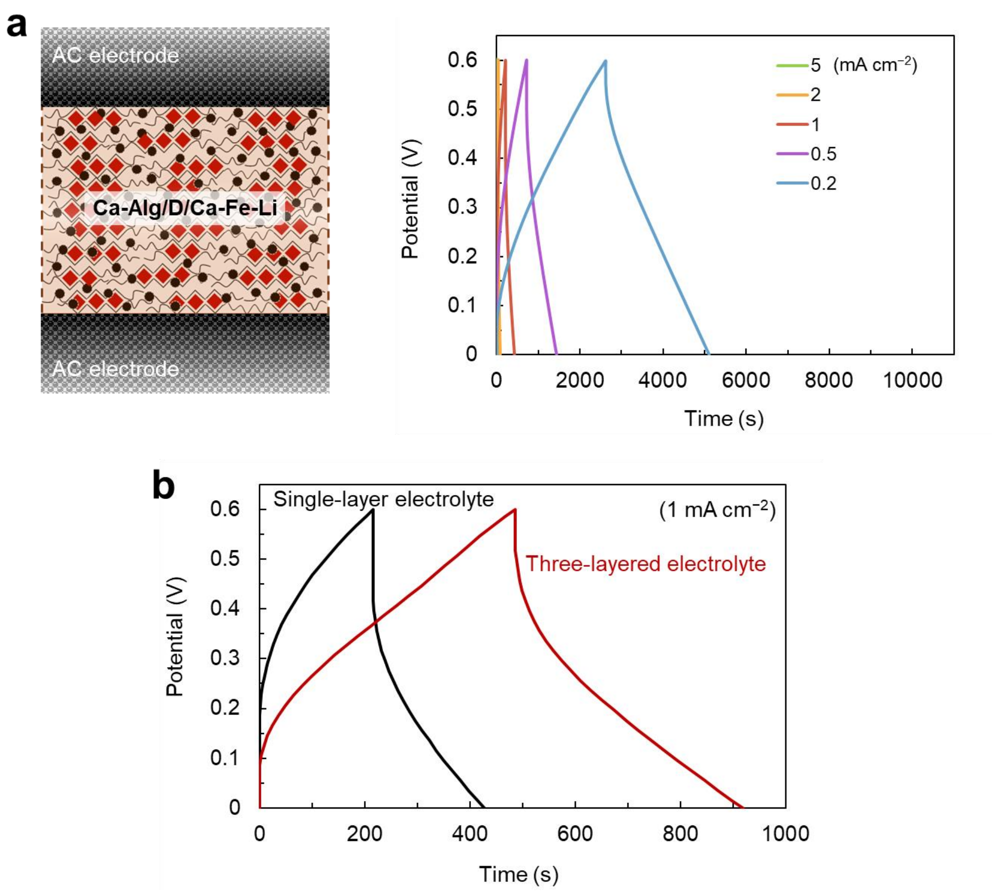

Supplementary Fig. 21. Supercapacitor composed of AC electrodes and single-layer hydrogel electrolyte. (a) Schematic of the supercapacitor and GCD curves at different current densities. The single-layer hydrogel electrolyte without PEDOT-contained layers resulted in lower supercapacitor performance compared to the supercapacitor composed of three-layered hydrogel electrolyte. (b) GCD curves showing the distinct difference between the two devices at a typical current density (1 mA cm^{-2}). The capacitance of the device composed of three-layered hydrogel electrolyte (778 mF cm^{-2}) was notably larger than that of the device composed of single-layer hydrogel electrolyte (492 mF cm^{-2}).

	Ionic conductivity (mS cm^{-1})
Ca-Alg/D/Ca-Fe-Water	0.0
Ca-Alg/D/Ca-Fe-Li	2.2

	Conductivity (mS cm^{-1})
Ca-Alg-PEDOT/D/Ca-Fe-Water	119.4
Ca-Alg-PEDOT/D/Ca-Fe-Li	168.4

Supplementary Table 1. Conductivity of hydrogels. The conductivity depends on the existence of mobile Li^+ ions and conducting polymer PEDOT.
